# The Influence of Diabetic Peripheral Neuropathy on Local Postural Muscle and Central Sensory Feedback Balance Control

**DOI:** 10.1371/journal.pone.0135255

**Published:** 2015-08-10

**Authors:** Nima Toosizadeh, Jane Mohler, David G. Armstrong, Talal K. Talal, Bijan Najafi

**Affiliations:** 1 interdisciplinary Consortium on Advanced Motion Performance (iCAMP) and Southern Arizona Limb Salvage Alliance (SALSA), Department of Surgery, College of Medicine, University of Arizona, Tucson, Arizona, United States of America; 2 Arizona Center on Aging, University of Arizona, Tucson, Arizona, United States of America; 3 Wound and Diabetic Foot Center, Department of Medicine, Hamad Medical Co., Doha, Qatar; Harvard Medical School, UNITED STATES

## Abstract

Poor balance control and increased fall risk have been reported in people with diabetic peripheral neuropathy (DPN). Traditional body sway measures are unable to describe underlying postural control mechanism. In the current study, we used stabilogram diffusion analysis to examine the mechanism under which balance is altered in DPN patients under local-control (postural muscle control) and central-control (postural control using sensory cueing). DPN patients and healthy age-matched adults over 55 years performed two 15-second Romberg balance trials. Center of gravity sway was measured using a motion tracker system based on wearable inertial sensors, and used to derive body sway and local/central control balance parameters. Eighteen DPN patients (age = 65.4±7.6 years; BMI = 29.3±5.3 kg/m^2^) and 18 age-matched healthy controls (age = 69.8±2.9; BMI = 27.0±4.1 kg/m^2^) with no major mobility disorder were recruited. The rate of sway within local-control was significantly higher in the DPN group by 49% (healthy local-control_slope_ = 1.23±1.06×10^-2^ cm^2^/sec, *P*<0.01), which suggests a compromised local-control balance behavior in DPN patients. Unlike local-control, the rate of sway within central-control was 60% smaller in the DPN group (healthy central-control_slope-Log_ = 0.39±0.23, *P*<0.02), which suggests an adaptation mechanism to reduce the overall body sway in DPN patients. Interestingly, significant negative correlations were observed between central-control rate of sway with neuropathy severity (*r*_*Pearson*_ = 0.65-085, *P*<0.05) and the history of diabetes (*r*_*Pearson*_ = 0.58-071, *P*<0.05). Results suggest that in the lack of sensory feedback cueing, DPN participants were highly unstable compared to controls. However, as soon as they perceived the magnitude of sway using sensory feedback, they chose a high rigid postural control strategy, probably due to high concerns for fall, which may increase the energy cost during extended period of standing; the adaptation mechanism using sensory feedback depends on the level of neuropathy and the history of diabetes.

## Introduction

It is estimated that some 382 million people had diabetes in 2013, and this number will increase 55% to 592 million people by 2035 [1]. High risk of fall has been reported in the diabetic population, with an overall incidence of 1.25 fall/person-year [2]. The risk of fall in this population increases with neuropathy (insensate feet), especially in older adults [2,3]. Although a direct objective predictor of falling risk has not yet been discovered, several studies have identified a strong association between poor postural balance and increased risk of falling [4,5]. Therefore, it is important to assess postural balance behaviors in persons with diabetic peripheral neuropathy (DPN).

Previous research has demonstrated more body sway during upright quiet standing in those with DPN, compared to those without [6,7]. To better understand the underlying mechanism that causes impaired balance in DPN individuals, we investigated the balance control mechanism based on its dependency on sensory feedback. Previous work has suggested that postural balance requires local postural muscle control (local-control) and higher central nervous sensory feedback cues (central-control) for the regulation of balance [8,9]. The local-control stage is known as a controlling mechanism that works without recruiting sensory feedback from visual, vestibular and/or somatosensory systems [8,10]. This mechanism is assumed to operate by setting an activity level required for postural muscles to control the short-term body fluctuations [8]. On the other hand, the central-control mechanism may be called into play in longer time-intervals of body sway, drawing upon sensory feedback to control balance [8,10,11]. To investigate the quality of balance control, separately, within the local- and central-control stages, the stabilogram diffusion analysis has been introduced. Briefly, in this approach, the trajectory of body center of mass on the ground surface (center of gravity; COG) is estimated and then used to derive the temporal displacement between adjacent COG data points to generate the stabilogram diffusion plot [8,12], and to define the local-control (short time-interval COG displacements) and the central-control (long time-interval COG displacements) stages [8].

Previous studies have reported marked reductions in strength, reflexive responses, and sensing function of lower extremity muscles in diabetic patients [13,14]. It is likely that these reductions in functioning of lower extremity muscles increase with neuropathy [15,16]. Therefore, the purpose of the current study was to investigate differences in the local- and central-control of balancing mechanism between DPN and healthy groups to determine alterations in balance performance due to impaired muscle functioning. We have hypothesized that DPN participants would demonstrate a compromised local-control balance performance compared to healthy controls, which would also influence the central-control strategy in DPN participants to adapt for this impairment. Furthermore, we assessed the association between DPN progression and alterations in local- and central-control of balance. Our second hypothesis was a deterioration in local-control balance by disease progression (i.e., the level of neuropathy and the history of diabetes), and consequently an increase in the level of adaptations in the central-control stage with the level of neuropathy and the history of diabetes.

## Materials and Methods

### Participants

Participants with type 2 diabetes and DPN were recruited from the Southern Arizona Limb Salvage Alliance (SALSA), at the University of Arizona. Healthy age-matched controls were recruited from a community-dwelling aging adults in a southwestern tertiary academic medical center, Tucson, Arizona. All participants were aged 55 years or above, with no major mobility disorder (able to perform 15-second upright standing). Participants were excluded if they had any clinically significant medical or psychiatric condition (including Dementia or Alzheimer’s), dizziness and nausea symptoms, or a sensation of a spinning motion in the head while standing upright, or a laboratory abnormality that could, in the judgment of the investigators, interfere with the ability to participate in the study. DPN participants were excluded if they had disorders other than diabetes leading to severe balance deficits (including stroke or Parkinson’s disease), an active foot ulcer, major foot deformity (e.g. Charcot neuroarthropathy), or major foot amputation. All participants signed a written informed consent form, specifically approved for this study by the University of Arizona institutional review board, and presented by trained clinical research coordinators.

### Neuropathy measurements and subjective questionnaires

Peripheral neuropathy was confirmed using the American Diabetes Association criteria based on insensitivity to 10-g Semmes-Weinstein monofilament [17,18]. Additionally, vibration perception threshold (VPT) scores were recorded to quantify the level of neuropathy with a cut-off of 25 mV as an indicator of neuropathy at recommended plantar foot sites. VPT was assessed at the great toe, fifth metatarsal, and heel of both feet using VPT Meter (Diabetica Solutions, Inc., San Antonio, Texas) [19]. Subjective measures for both groups included fall status (history of fall within one year prior to measurement) and fear of falling based on the Falls Efficacy Scale-International (FES-I) questionnaire [20].

### Balance measurements

**Fig 1:**
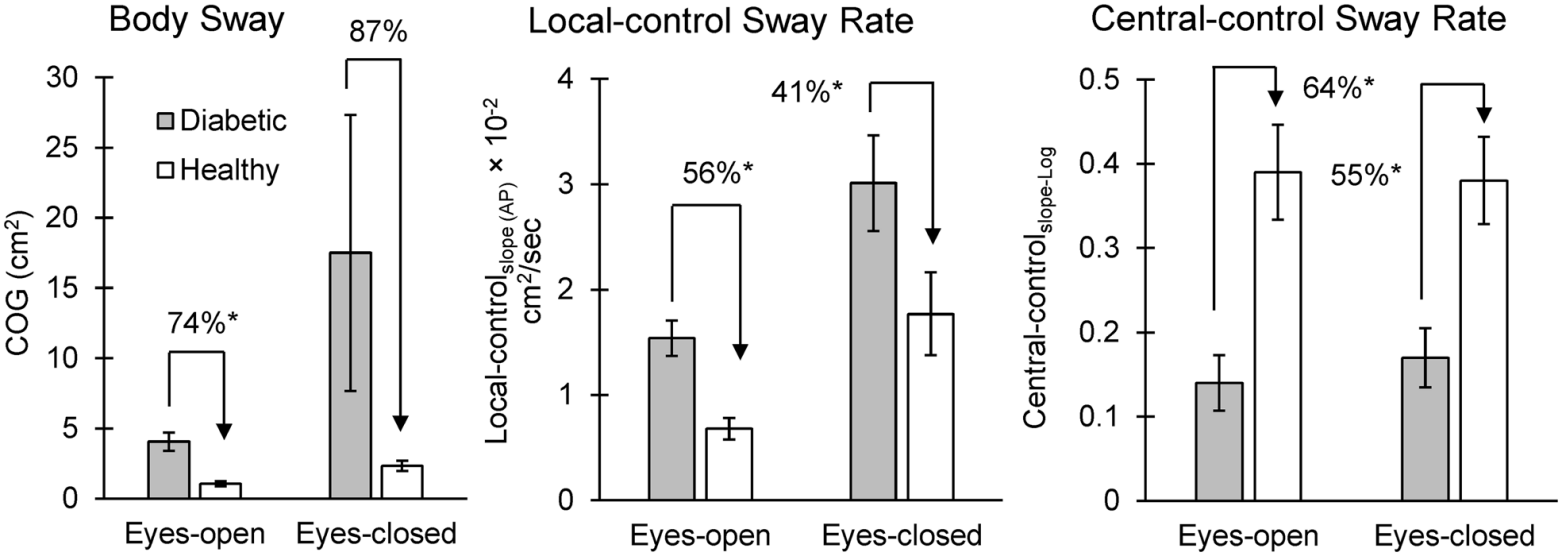
Differences in balance parameters among DPN and healthy groups, in eyes-open and eyes-closed conditions. Local/central control balance parameters with highest effect sizes on average among eyes-open and eyes-closed conditions are illustrated. Significant differences are highlighted with asterisks.

Each participant performed two 15-second trials of balance assessment, within which participants stood upright with their feet together as close as possible without touching each other, and with their arms crossed across their chest. Participants were instructed to keep their eyes open (no target was specified) in the first trial, and to keep their eyes closed in the subsequent trial. Two sensors (Fig 1), each including a triaxial accelerometer and a triaxial gyroscope were used to estimate three-dimensional angles of the ankle and hip joints (BalanSens, BioSensics LLC, MA, USA). The COG was estimated for each trial using a two-link inverted pendulum following identical procedures reported in our earlier studies [12,21,22]. Briefly, the two-link model was used to calculate anterior-posterior (AP) and medial-lateral (ML) angles of legs (lower link-ankle rotation) and upper-body (upper link-hip rotation). Using participants’ anthropometric data, the mass and center of mass were estimated for each link (see [21] for calculation procedure). A wavelet transform band-pass filter (Coiflet 5—cutoff frequency of 0.06–30 Hz) was used to reduce the noise related to skin movement.

Traditional body sway parameters from COG plots include: COG _(AP)_ sway, COG _(ML)_ sway, and COG sway. The ranges of sway in the AP and ML directions were defined as COG _(AP)_ sway and COG _(ML)_ sway, respectively, after excluding outliers as described in previous work [21,23]. The COG sway (i.e., total sway) was then calculated by multiplying the range of motion in AP and ML directions (i.e., COG sway = COG _(AP)_ sway × COG _(ML)_ sway).

To assess the performance of local- and central-control balance strategies, we used the stabilogram diffusion analysis as described by Collins et al. [8,11]. This method was used to estimate rate of body sway in short time-intervals (the local postural muscle control; local-control) and long time-intervals (the higher central nervous system using sensory cueing postural control; central control). The hypothesis beyond this method is that higher rate of body sway in short time-intervals (e.g., < 1.5 seconds) reflects inability of postural muscles in maintaining the required moment for balance; while, higher rate of sway in long time-intervals (e.g., > 2.5 seconds) suggests inability of postural muscles or a compromised sensory regulation for maintaining balance. Extracted parameters from this method included: local-control_slope_, central-control_slope-Log_, and local-control_time-interval_. (See Appendix A for details of parameter estimations). All parameters were reported in AP, ML, and for the total sway. Local-control_slope_ represent the rate of body sway within the local-control stage in normal scales. Central-control_slope-Log_ represent the rate of body sway within the central-control stage in logarithmic scale. Logarithmic scales were calculated here to better highlight balance impairments within long time-intervals (central-control) as suggested by previous work [24]. Local-control_time-interval_ represents the maximum time interval in which the central-control mechanism is added to the local-control.

### Statistical analysis

Separate analyses of covariates (ANCOVAs) were used to compare the effect of DPN on balance parameters, considering age, body mass index (BMI), and gender as covariates; Cohen’s effect size was calculated. Correlations between balance parameters with neuropathy and history of disease were assessed using linear regression-ANOVA models, considering age, BMI, and gender as adjusting variables; correlation results were reported as Pearson correlations (*r*). Pearson correlations were also used to assess associations between stabilogram diffusion parameters with age, height, weight, and BMI. Summary results are presented as means (standard deviation—SD). All analyses were done using JMP (Version 10, SAS Institute Inc., Cary, NC), and statistical significance was concluded when *p* ≤ 0.05.

## Results

### Participants

Thirty-six participants (18 with confirmed DPN and 18 healthy controls) were recruited and completed all tests. Mean age and BMI of DPN participants were 65 ± 8 years and 29.3 ± 5.4 kg/m^2^, respectively; corresponding values were 69 ± 3 years and 27.0 ± 4.1 kg/m^2^ for the healthy controls. The history of DPN and the VPT score were 19 ± 11 years (from one to 35 years) and 34.6 ± 7.0 mV (from 26 to 52 mV), respectively, for DPN participants. As expected a higher fear of falling (as measured by FES-I) and a larger number of fallers (based on fall history) were determined among DPN participants compared to healthy controls (*P* < 0.05). Demographic details are reported in Table 1 (primary-level data in S1 Table).

**Table 1:** Demographic information of participants. Significant differences are highlighted with asterisks.

	Diabetic	Healthy	*P*-value
**Number (% of total)**	18 (50%)	18 (50%)	-
**Male, n (% of the group)**	11 (61%)	7 (39%)	0.12
**Age, years**	65 ± 8	69 ± 3	0.06
**Stature, cm**	174.0 ± 10.8	161.9 ± 4.5	<.0001*
**Body mass, kg**	88.9 ± 18.6	71.2 ± 13.1	<.01*
**BMI, kg/m** ^**2**^	29.3 ± 5.4	27.0 ± 4.1	0.16
**FES-I, 16–64 scale**	30.7 ± 12.2	20.4 ± 3.2	<.01*
**Fallers, n (% of the group)**	10 (56%)	4 (22%)	0.04*
**VPT, mV**	34.6 ± 7.0	-	-
**Diabetes history, years**	19 ± 11	-	-

BMI = body mass index

FES-I = Falls Efficacy Scale-International

VPT = vibration perception threshold

### Differences in balance behaviors between DPN and healthy groups

As expected, body sway (i.e., COG) in the DPN group was significantly higher than controls, respectively by 74% and 87% in eyes-open and eyes-closed conditions (Tables 2 and 3, and Fig 1; primary-level data in S1 Table). Local-control_time-interval_ was not significantly different among the two groups (Tables 2 and 3, and Fig 1), which means that the longest time-interval during upright standing that sensory cueing are added to help maintaining balance was approximately the same for both groups. However, local-control_slope_, rate of body sway within the local-control stage, was significantly higher in DPN group by 56% in eyes-open and 41% in eyes-closed conditions (Tables 2 and 3, and Fig 1). Interesting, unlike the local-control stage, central-control_slope-Log_ was 64% and 55% smaller in the DPN group compared to healthy controls in eyes-open and eyes-closed conditions, respectively (Tables 2 and 3, and Fig 1). This suggests that the rate of body sway was smaller in DPN patients when receiving cuing from sensory feedback.

**Table 2:** Mean, SD, and ANCOVA results for body sway and local/central control parameters in the eyes-open condition for DPN and healthy groups. Significant differences are highlighted with asterisks.

**Body Sway Parameters**	**Diabetic**	**Healthy**	***P*-value**	**Effect Size**
**COG**_**(AP)**_ **cm**	3.13 ± 1.16	1.71 ± 0.81	<.01*	1.43
**COG**_**(ML)**_ **cm**	1.20 ± 0.47	0.60 ± 0.28	<.01*	1.57
**COG cm** ^**2**^	4.06 ± 2.79	1.06 ± 0.76	<.01*	1.46
**local/central Control Balance Parameters**	**Diabetic**	**Healthy**	***P*-value**	**Effect Size**
**Local-control**_**slope (AP)**_ **× 10** ^**−2**^ **cm** ^**2**^ **/sec**	2.07 ± 1.04	0.92 ± 0.86	<.01*	1.21
**Central-control**_**slope-Log (AP)**_	0.14 ± 0.17	0.43 ± 0.22	<.01*	1.45
**Local-control**_**time-interval (AP)**_ **sec**	2.35 ± 1.01	1.96 ± 0.66	0.65	0.46
**Local-control**_**slope (ML)**_ **× 10** ^**−2**^ **cm** ^**2**^ **/sec**	1.23 ± 0.65	0.45 ± 0.39	<.01*	1.47
**Central-control**_**slope-Log (ML)**_	0.19 ± 0.15	0.39 ± 0.20	<.01*	1.12
**Local-control**_**time-interval (ML)**_ **sec**	2.65 ± 0.90	2.16 ± 0.67	0.08	0.63
**Local-control**_**slope**_ **× 10** ^**−2**^ **cm** ^**2**^ **/sec**	1.54 ± 0.71	0.68 ± 0.44	<.01*	1.58
**Central-control**_**slope-Log**_	0.14 ± 0.14	0.39 ± 0.24	<.01*	1.30
**Local-control**_**time-interval**_ **sec**	2.37 ± 1.02	2.08 ± 0.87	0.35	0.30

COG = center of gravity

AP = anterior-posterior

ML = medial-lateral

**Table 3:** Mean, SD, and ANCOVA results for body sway and local/central control parameters in the eyes-closed condition for DPN and healthy groups. Significant differences are highlighted with asterisks.

**Body Sway Parameters**	**Diabetic**	**Healthy**	***P*-value**	**Effect Size**
**COG**_**(AP)**_ **cm**	5.42 ± 4.96	2.82 ± 1.39	<.05*	0.72
**COG**_**(ML)**_ **cm**	1.96 ± 1.61	0.80 ± 0.35	0.01*	0.99
**COG cm** ^**2**^	17.50 ± 41.68	2.34 ± 1.56	0.12	0.52
**local/central Control Balance Parameters**	**Diabetic**	**Healthy**	***P*-value**	**Effect Size**
**Local-control**_**slope (AP)**_ **× 10** ^**−2**^ **cm** ^**2**^ **/sec**	4.67 ± 3.09	2.01 ± 1.99	0.02*	1.02
**Central-control**_**slope-Log (AP)**_	0.15 ± 0.11	0.41 ± 0.20	<.001*	1.67
**Local-control**_**time-interval (AP)**_ **sec**	1.76 ± 0.83	1.97 ± 1.08	0.56	0.23
**Local-control**_**slope (ML)**_ **× 10** ^**−2**^ **cm** ^**2**^ **/sec**	2.55 ± 2.32	0.99 ± 0.83	0.04*	0.91
**Central-control**_**slope-Log (ML)**_	0.16 ± 0.14	0.34 ± 0.23	0.03*	0.96
**Local-control**_**time-interval (ML)**_ **sec**	2.20 ± 0.74	2.11 ± 0.79	0.76	0.13
**Local-control**_**slope**_ **× 10** ^**−2**^ **cm** ^**2**^ **/sec**	3.01 ± 1.93	1.77 ± 1.67	0.08	0.73
**Central-control**_**slope-Log**_	0.17 ± 0.15	0.38 ± 0.22	0.02*	1.09
**Local-control**_**time-interval**_ **sec**	1.54 ± 0.84	1.90 ± 1.09	0.30	0.37

COG = center of gravity

AP = anterior-posterior

ML = medial-lateral

### Alterations in balance parameters with neuropathy, history of DPN, and demographic information

Significant negative correlations were observed between the VPT score and central-control_slope-Log (AP)_ (*P* < 0.01; *r* = -0.81), central-control_slope-Log (ML)_ (*P* = 0.05; *r* = -0.65), and central-control_slope-Log_ (*P* < 0.001; *r* = -0.85) within the eyes-closed condition. Similarly, significant correlations were determined between disease history and central-control_slope-Log (AP)_ in eyes-open (*P* = 0.01; *r* = -0.71) and eyes-closed (*P* = 0.02; *r* = -0.58) conditions. These correlations represent an overall reduction in the rate of body sway within the central-control stage with the VPT score and disease history. On the other hand the rate of sway within local-control stage increased with neuropathy severity and disease history, approaching significance in our sample (e.g., for local-control_slope (ML)_ *P* = 0.07; *r* = 0.57). No significant correlation was found between stabilogram diffusion parameters with age (*r* ≤ 0.45), height (*r* ≤ 0.54), weight (*r* ≤ 0.47), or BMI (*r* ≤ 0.51) in either DPN or healthy groups (*p* > 0.06).

## Discussion

### Local-control balance deterioration in DPN patients

The results of this study suggest that DPN affects the amount of body sway in short time-intervals or local-control stage when comparing with healthy controls. This could be related to inability of lower extremity and postural muscles to provide sufficient activity level and joint stiffness in patients with DPN. It has been previously claimed that the strength of lower extremity muscles drops with neuropathy. Anderson et al. reported 17% and 14% less strength in ankle flexor and extensor muscles of DPN patients, respectively, when compared to age-matched healthy controls [13]. The reduction in muscle strength has been associated with increased glucose concentration, and potentially reduced glucose uptake and hyperglycemia in muscles, which can lead to lower levels of physical activity [25,26]. Muscle strength is the key component for short time-interval balance control by setting an activity level in postural muscles based on muscle loading, and lack of adequate muscle strength can compromise the short time-interval (local-control) balance mechanism [27].

Compromised reflexive responses of lower extremity muscles have also been associated with DPN [28–30]. It has been hypothesized that reflexive responses of lower extremity muscles act in an opposing/cancelling mechanism during the upright quiet standing. For example, when a stretch reflex is excited in ankle flexor muscles, the stretch reflex in the opposing extensor muscles is inhibited [31]. Previous studies have reported a completely diminished, or delayed and reduced-amplitude reflexive responses of skeletal muscles in diabetic patients compared to healthy controls [14,32,33]. Although the electromyography data was not available within the current study, we believe that lack of efficient reflexive responses of the lower extremity muscles may be another reason for the increased rate of body sway within the local-control stage. However, future studies are required to confirm the effect of reflex deterioration in balance performance among DPN patients.

In addition to deterioration of reflexive responses and reduction in strength, previous work suggested that spindle sensation of muscles (i.e., position, velocity, and force sensation) also compromises with DPN. van Deursen et al. used a tracking performance protocol with and without vibration to assess the muscle spindle function of the ankle [16]. Their results demonstrated that DPN degrades muscle sensory function, as much as half of the healthy controls performance, which may cause poorer balance in DPN patients [16]. In a general posture regulation model developed by Nashner, it has been suggested that muscle spindle sensation and force feedback regulate at the spinal level [31]. On the other hand, vestibular, extroceptive, and visual feedback regulate at higher levels of the nervous system [31]. Based on this model and all previous evidence, we infer here that lack of muscle strength, compromised reflexive responses and sensory performance of postural muscles lead to impaired local-control balance behaviors during upright standing.

### Central-control balance alteration in DPN patients

Unlike local-control, our results demonstrated small sway rate within the central-control stage in DPN group, even smaller than healthy controls during both eyes-open and eyes-closed conditions. Similar findings were reported by Collins et al. when they compared healthy young and older adults. They discovered smaller central-control_slope-Log_ in the older group compared to younger participants, which was interpreted as an adaptation to compensate for a worsen local-control strategy with aging [24]. Within the current study setting, however, it is impossible to make a direct conclusion regarding the benefits of this central-control adaptation with DPN; the balance alteration in DPN participants within the central-control stage may be explained either as a rigid compensatory strategy after receiving sensory cueing probably due to high concern for falls or as an adaptation in DPN group by improving the sensory cueing postural control to compensate for deterioration in local-control balance. In case of first interpretation, the high rigidity within central-control postural strategy may reduce the ability of DPN participants to adapt to irregular surfaces and their quick response to any postural alterations [34], or may increase the energy cost during upright standing and cause fatigue in postural muscles. In case of second interpretation, sensory cueing may improve in DPN patients to compensate for influenced postural muscle balance control, by reducing the rate of body sway within the central-control stage. Additional experimental studies are, therefore, required by implementing alternative postural control settings (e.g., irregular surfaces or sudden perturbation) to further explore these findings.

To assess changes in postural control strategies with disease severity, additional analyses were performed to explore the association between local- and central-control performance with the level of neuropathy and history of DPN. Interestingly, we observed that by increasing the VPT score and history of DPN, the rate of body sway within the central-control stage decreases. This reduction in the rate of body sway within central-control balance was mainly observed in the eyes-closed condition. Accordingly, it can be concluded that sensory units other than visual feedback may involve in the central-control adaptation among the DPN group; however, this hypothesis requires further investigation of the effect of sensory feedback mechanisms in order to separately assess vestibular, extroceptive, and visual balance regulation performance.

### Limitations and summary of findings

A convenient sample of DPN and healthy controls were recruited for the current study, and, therefore, the current results, while encouraging, should be confirmed in a larger sample size. However, we managed to recruit DPN participants with different history of diabetes and covered a wide range of neuropathy levels to better associate balance parameters with the disease severity. Moreover, in the implemented approach, two distinct stages of local-control and central-control are considered, which means that at one specific time interval sensory feedback is added to control the balance. However, Newell et al. [9] criticized that the different sources of sensory feedback work continuously at different time intervals. Although, the current findings provide a better understanding of performance of local- and central-control balance strategies, due to these limitations, results of the current study should be interpreted cautiously, and confirmed with other experimental measurement and modeling approaches.

In summary, our findings from the current research provide insights regarding balancing strategies in DPN patients based on dependency on postural muscles and sensory feedback. For the first time, using stabilogram diffusion analysis, we were able to explain the large amount of body sway in DPN patients during quiet standing according to local- and central-control balancing behaviors. First, we concluded that in the lack of sensory feedback cueing, rate of sway was on average ~49% higher in DPN participants compared to healthy controls in both eyes-open and eyes-closed conditions. This compromised local-control balance control may be explained by the poor local muscle control response (i.e., reduced muscle strength, deteriorated reflexive responses, and impaired muscle spindle performance in sensing). Second, we observed that after receiving central cueing, rate of body sway dropped substantially even beyond the range of healthy controls balance behaviors, suggesting an adaptation mechanism in DPN patients that depends on history of disease and the level of neuropathy. Overall, these new findings introduced a new approach of postural balance assessment, which may be helpful in better understanding of balance alterations with DPN, and ultimately implement more efficient strategies to improve balance performance using exercise routines or medical treatments.

## Appendix A

**Fig 2:**
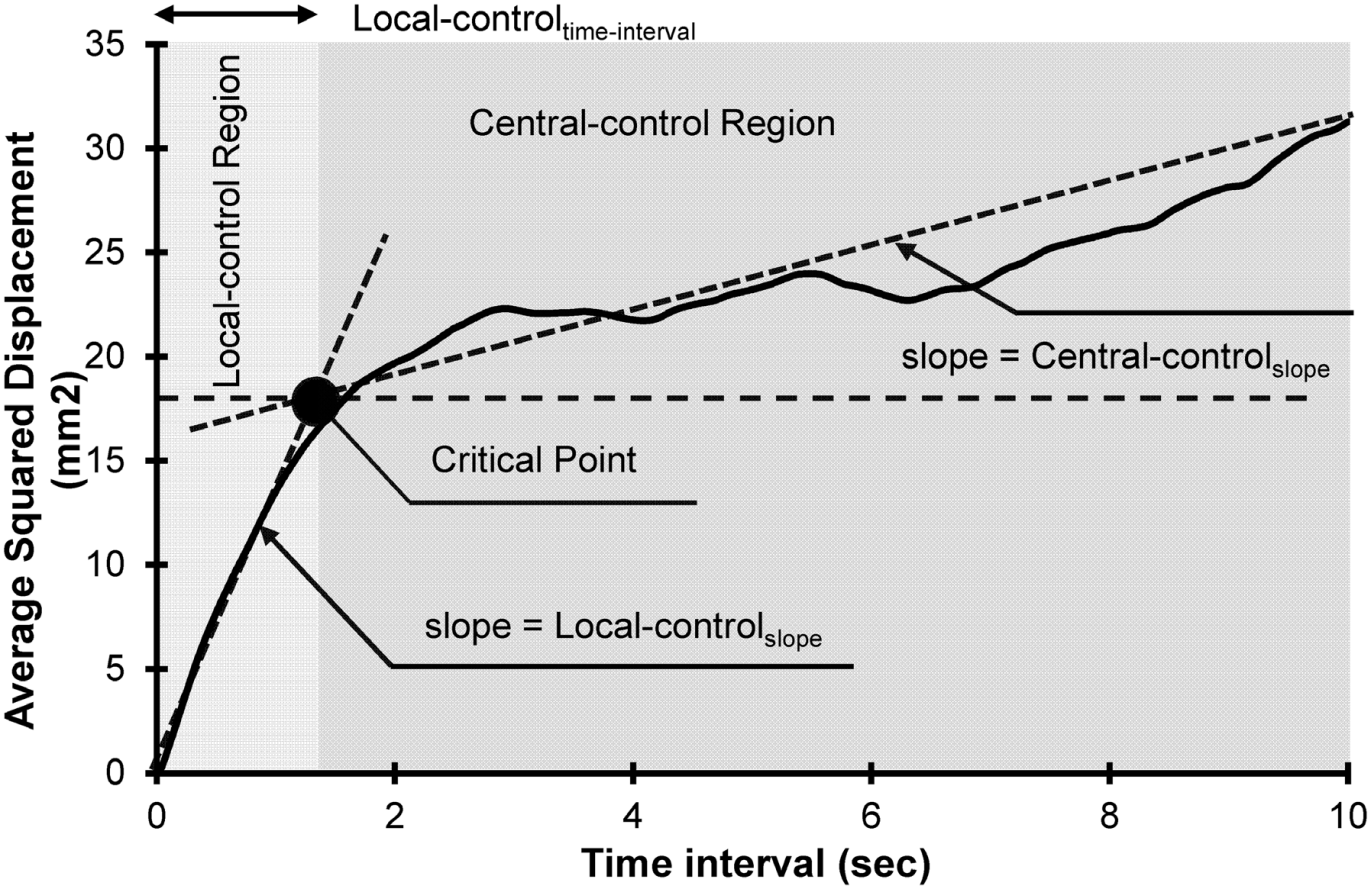
A sample of stabilogram diffusion plot from a healthy control participant. The figure illustrates two separate regions, which are local-control and central-control regions. Fitted line slopes in each region (local-control_slope_ and central-control_slope_) indicates the rate of change in magnitude of sway as a function of time-interval. The critical point (local-control_time-interval_) is the intersection of these two lines.

To identify local-control and central-control strategies, the square of displacement (Δ*r*_*i*_)^2^ between successive COG data points separated in time by a specified time-interval (Δ*t*) were calculated. The squared displacements (Δ*r*_*i*_)^2^ were then averaged over the specified time-interval (Δ*t*), ranging from 0 to 10 seconds (0 ≤ Δ*t* ≤ 10 sec) to provide a plot of mean square COG displacement (Δ*r*)^2^ versus Δ*t* according to the following formula:

$$<\Delta r^{2}{>}_{\Delta t}=\frac{\sum_{i=1}^{N-m}{\left(\Delta r_{i}\right)}^{2}}{\left(N-m\right)}$$ (1)

where, N is the number of data points for the first 10 seconds of COG data series, and for a given Δ*t*, *m* is the number of data intervals. Using this approach a stabilogram diffusion plot is developed (Fig 2), which represents square value of COG displacement (Δ*r*)^2^ as a function of time-interval (Δ*t)*. Two distinguishable regions exist in this plot, named as short time-interval (local-control) and long time-interval (central-control) regions, which are separated by identifying the critical time-interval (local-control_time-interval_: critical time-interval (Δ*r*_*c*_). Local-control_time-interval_ is estimated by identifying two best linear fits, which represent, respectively, the short time-interval linear fit (fitted on short time-interval data points) and long time-interval linear fit (fitted on long time-interval data points) of the stabilogram diffusion plot as illustrated in Fig 2. The slope of these two fits were then calculated to represent estimations of the rate of sway as a function of time-interval during local-control (local-control_slope_) and central-control (central-control_slope_) conditions. Similar to local-control_slope_ and central-control_slope_, the scaling exponents, local-control_slope-Log_ and central-control_slope-Log_ were calculated from short time-interval and long time-interval slopes of the log-log stabilogram diffusion plot. Based on fractional Brownian theory, the following equation exist between squared of displacement and time-interval:

$${\left(\Delta r\right)}^{2}=\Delta t^{2H}$$ (2)

where, scaling exponent H can be a number between 0 to 1, and was estimated here using slopes of the log-log plot of the mean squared displacements versus time-interval (i.e., log-log stabilogram diffusion plot); central-control_slope-Log_ in the current study represent scaling exponent H within the long time-interval region. The scaling exponent determines the correlation between the past and future increments. If H > 0.5, the COG moving in a particular direction, will tend to continue in the same direction. On the other hand, for H < 0.5 the correlation between past and future movement is negative. Based on previous work [24], in regions with small scaling exponent (H < 0.5 –the central-control region) differences between normal and impaired balance behavior can be better highlighted using logarithmic scale slopes compared to diffusion plot slopes. (readers are refer to [8,35] for more details regarding fractional Brownian theory).

## Supporting Information

S1 Table Primary-level data. (XLSX)
